# Calcium Electroporation: Evidence for Differential Effects in Normal and Malignant Cell Lines, Evaluated in a 3D Spheroid Model

**DOI:** 10.1371/journal.pone.0144028

**Published:** 2015-12-03

**Authors:** Stine Krog Frandsen, Laure Gibot, Moinecha Madi, Julie Gehl, Marie-Pierre Rols

**Affiliations:** 1 Center for Experimental Drug and Gene Electrotransfer, Department of Oncology, Copenhagen University Hospital Herlev, 2730 Herlev, Denmark; 2 CNRS, Institut de Pharmacologie et de Biologie Structurale, Toulouse, France; 3 Université de Toulouse, UPS, Institut de Pharmacologie et de Biologie Structurale, Toulouse, France; University of California at Berkeley, UNITED STATES

## Abstract

**Background:**

Calcium electroporation describes the use of high voltage electric pulses to introduce supraphysiological calcium concentrations into cells. This promising method is currently in clinical trial as an anti-cancer treatment. One very important issue is the relation between tumor cell kill efficacy–and normal cell sensitivity.

**Methods:**

Using a 3D spheroid cell culture model we have tested the effect of calcium electroporation and electrochemotherapy using bleomycin on three different human cancer cell lines: a colorectal adenocarcinoma (HT29), a bladder transitional cell carcinoma (SW780), and a breast adenocarcinoma (MDA-MB231), as well as on primary normal human dermal fibroblasts (HDF-n).

**Results:**

The results showed a clear reduction in spheroid size in all three cancer cell spheroids three days after treatment with respectively calcium electroporation (p<0.0001) or electrochemotherapy using bleomycin (p<0.0001). Strikingly, the size of normal fibroblast spheroids was neither affected after calcium electroporation nor electrochemotherapy using bleomycin, indicating that calcium electroporation, like electrochemotherapy, will have limited adverse effects on the surrounding normal tissue when treating with calcium electroporation. The intracellular ATP level, which has previously been shown to be depleted after calcium electroporation, was measured in the spheroids after treatment. The results showed a dramatic decrease in the intracellular ATP level (p<0.01) in all four spheroid types—malignant as well as normal.

**Conclusion:**

In conclusion, calcium electroporation seems to be more effective in inducing cell death in cancer cell spheroids than in a normal fibroblast spheroid, even though intracellular ATP level is depleted in all spheroid types after treatment. These results may indicate an important therapeutic window for this therapy; although further studies are needed *in vivo* and in patients to investigate the effect of calcium electroporation on surrounding normal tissue when treating tumors.

## Introduction

Electroporation or electropermeabilization is a method to generate transient permeabilization of the cell membrane by applying short, intense electric pulses [1]. The method can be used to facilitate transport of normally non-permeant ions or molecules into most cell types [2]. This method is used in the clinic in combination with chemotherapeutic drugs (electrochemotherapy) where the effect of the drug is increased drastically [2–5]. Due to common side effects of most chemotherapeutic drugs including bleomycin [6]; it is appealing to be able to use non-toxic molecules or ions that will induce cell death, when they are internalized into the cells in high concentration by electroporation. Calcium is a tightly regulated ubiquitous second messenger that is involved in regulation of transcription, metabolism, proliferation, apoptosis, necrosis, and many other cellular processes [7–9]. Calcium electroporation, where calcium is introduced into the cell cytosol by electroporation, has previously been shown to efficiently induce cell death *in vitro* and *in vivo* associated with acute ATP depletion [10], using electroporation parameters similar to those used for electrochemotherapy [11]. Calcium electroporation is currently in clinical trial (ClinicalTrials.gov ID- NCT01941901) for cutaneous metastasis where the response of the treatment will be compared with the response of electrochemotherapy using bleomycin, which is a clinically used anti-cancer treatment [12,13]. Calcium electroporation has been proven efficient in cell death of cancer cells but the effect on a broader range of tumor cell lines and on normal cells has not previously been investigated. We have tested the effect of calcium electroporation and electrochemotherapy using bleomycin in four different human cell spheroids (three cancer cell lines and one normal primary cell type) in order to investigate any difference in sensitivity between the different cell types. We chose using 3D spheroids as an *in vivo*-like model of tumors, a system which has previously been used for electroporation and electrochemotherapy studies [14,15].

## Materials and Methods

### Chemicals

Propidium iodide (PI; Sigma-Aldrich), bleomycin sulfate (Bleo; Merck-Millipore), and calcium chloride (Ca; SAD, Denmark).

### Cell culture

Three human cancer cell lines and one normal human cell type were used in this study. HT29 cells (ATCC #HTB-38), from a human colorectal adenocarcinoma, were grown in RPMI-1640 culture medium (Gibco, Invitrogen). SW780 (kindly provided by Dr. Lars Dyrskjøt Andersen, Department of Molecular Medicine, Aarhus University Hospital, Skejby, Denmark), a human bladder transitional cell carcinoma [16], MDA-MB-231 (ATCC #HTB-26), a human breast adenocarcinoma, and human dermal fibroblasts HDF-n (ScienCell #2310) were grown in DMEM culture medium (Gibco, Invitrogen). All cells grew with 10% fetal calf serum (Gibco, Invitrogen) and 100 U/ml penicillin and 100 μg/ml streptomycin, and all tested negative for mycoplasma using MycoAlert mycoplasma detection kit (Lonza). Cells were maintained at 37°C in a humidified atmosphere containing 5% CO_2_.

### Spheroid formation

Spheroids were produced with a non-adherent technique as previously described [14]. Briefly, 5000 cells were seeded in ultra-low attachment 96-well plates (Corning, Fisher Scientific) that were centrifuged 5 min at 4°C at 300 g. Spheroids were cultivated for 5 days at 37°C in a humidified atmosphere containing 5% CO_2_. Day of treatment was denoted as day 0. The normal dermal fibroblast spheroids were smaller as primary normal cells do not grow as aggressively as cancer cell lines.

### Electroporation conditions

One spheroid at the time was incubated 5 min in 100 μl of HEPES buffer (10 mM HEPES, 250 mM sucrose, and 1 mM MgCl_2_ in sterile water) with added drug before placed between two stainless steel, flat, parallel electrodes (0.4 cm between electrodes) in 100μl of HEPES buffer with added drug and 8 square-wave pulses of 100 μs, 1 Hz, and 1000 V/cm or 5000 V/cm (applied voltage to electrode distance ratio) were delivered by the Electro cell S20 generator (βTech, France) at room temperature. After treatment, spheroids were rinsed in HEPES buffer and cultivated in cell culture media before analysis. Membrane permeabilization was assessed by pulsing spheroids in the presence of 100 μM propidium iodide after 5 min incubation (1000 V/cm) followed by fluorescence imaging as in [15]. The 1000 V/cm parameters (applied voltage to electrode distance ratio) are the same as the ones used in our previous *in vivo* study [10] and used in the clinical setting [12].

### Spheroid growth experiment

After harvesting, spheroids were washed in HEPES buffer and incubated for 5 min in respectively HEPES buffer (control), 168 mM CaCl_2_ (as used before [10]), or 1 mM bleomycin (as used before [15]) before electric pulse application or not.

Growth of spheroids was followed by light microscopy before treatment and at day 2, 3, and 4 after treatment using a Leica DMIRB microscope coupled to a coolSNAP HQ camera (Roper Scientific, Photometrics) and size determined using Image J software (NIH, Bethesda, USA). The normalized area was expressed as the ratio of the post-treatment spheroid 2D area compared to the area before treatment as in [14,15].

Differences in normalized areas in the 6 treatment groups were evaluated as repeated measurements, validated and analysed with an exponential decrease model with Bonferroni correction using SAS software version 9.2. ‘Group’, ‘days’ and ‘spheroid’ were considered factors and baseline level of spheroid area as covariant.

### Viability

Four days after treatment, spheroids were incubated for 30 min at 37°C with 2 μM Calcein-AM and 4 μM EthD-1 (LIVE/DEAD viability/cytotoxicity kit, Invitrogen). In living cells, active intracellular esterase cleaves the calcein-AM to intensely fluorescent calcein, which is retained within cells with membrane integrity. Fluorescence was observed using a Leica DMIRB microscope coupled to a coolSNAP HQ camera (Roper Scientific, Photometrics). In order to compare pictures, fluorescence intensities were normalized using the maximum value, using Image J software (NIH, Bethesda, USA).

MDA-MB231 spheroids could not be tested since the spheroids were destroyed after both calcium electroporation and electrochemotherapy using bleomycin, and therefore transfer of the spheroids at day 4 to do live/dead staining could not be performed.

### ATP assay

Spheroids of the four cell types were electroporated as described above with 168 mM calcium. Spheroids electroporated with HEPES buffer, non-electroporated spheroids incubated with 168 mM calcium and untreated spheroids were used as controls. Additionally, one group of spheroids was exposed to very high voltage electroporation (8 pulses of 100 μs, 5000 V/cm, and 1 Hz); the highest electric field possible to apply with the used equipment and with the electrode geometry employed. This was done with the aim of examining cell death but results showed that this high electric field was not sufficient for complete cell death. Spheroids were lysed 1, 4, 24, and 72 hours after treatment with 100 μL of lysis buffer (50 mM TRIS pH 8, 1 mM EDTA, 0.5% Tween20), and ATP content determined by adding 100 μL rL/L Reagent (ENLITEN ATP Assay, Promega) and measuring light emission using a luminometer (Clariostar, BMG). Difference in ATP level after different treatments was assessed by 2-way ANOVA with post least-squares-means test with Bonferroni correction.

## Results and Discussion

**Fig 1:**
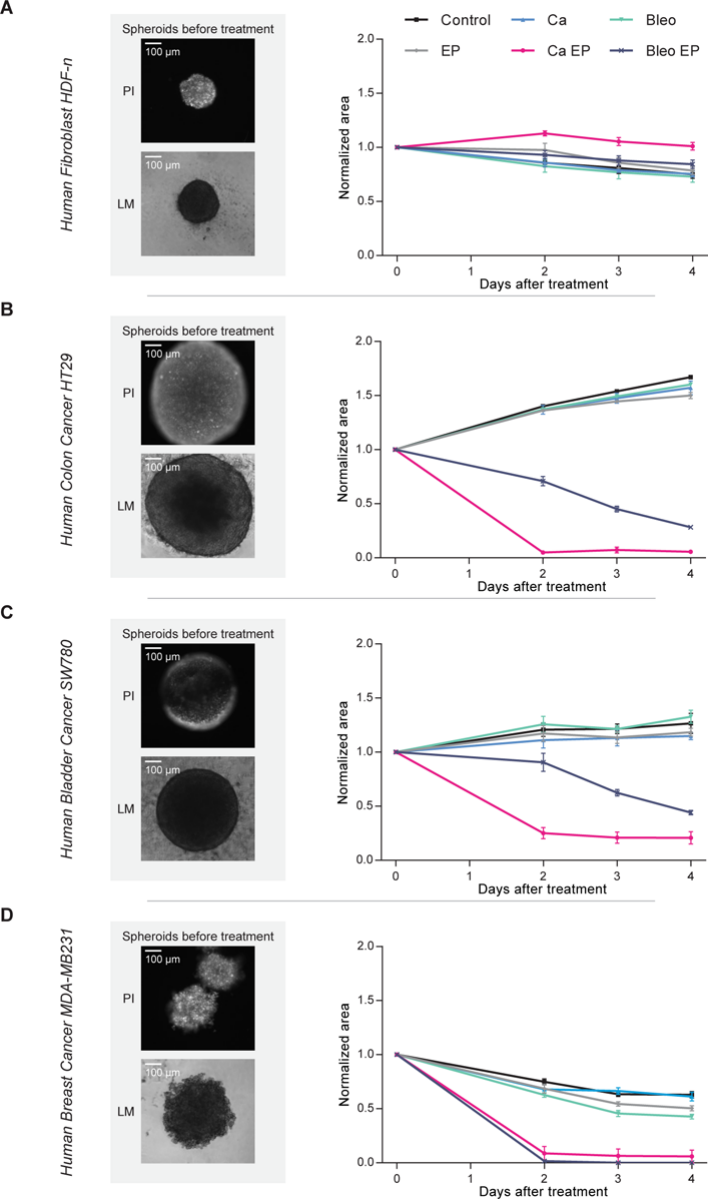
Spheroid size. Size measurements of human normal dermal fibroblast (A), colon cancer (B), bladder cancer (C), and metastatic breast cancer (D) spheroids. Left panel: representative fluorescence microscopy images of a spheroid just after electroporation (8 pulses of 100 μs, 1000 V/cm, and 1 Hz) in buffer containing propidium iodide (PI) to visualize electropermeabilized cells and light microscopy images of another spheroid just before treatment (LM). Right panel: Growth curves of the spheroids after treatment with respectively 168 mM calcium (Ca), 1 mM bleomycin (Bleo), electroporation (EP), 168 mM calcium electroporation (Ca EP), electrochemotherapy using 1 mM bleomycin (Bleo EP), and untreated controls (Control). Measurements performed before treatment and at day 2, 3, and 4. Spheroid size is normalized to the size before treatment, means +/- SD, n = 5–6.

Calcium electroporation seemingly had no effect on the size of the human fibroblast spheroids compared with untreated controls, but efficiently reduced the size of the three different cancer cell spheroids compared with untreated controls (p<0.0001 three days after treatment), at least as efficiently as electrochemotherapy using bleomycin (p<0.0001 three days after treatment; Fig 1). Although the effect of calcium electroporation appears to be superior to the effect of electrochemotherapy using bleomycin in two of the cancer cell spheroids (HT29 and SW780), this could be due to the chosen concentrations (168 mM CaCl_2_ as previously used *in vivo* [10] and 1 mM bleomycin as previously used in spheroids [15]).

As seen in Fig 1, despite seeded at the same density, the spheroids differ in size on the day of treatment with the fibroblast spheroids being the smallest (182 +/- 9 μm in diameter) followed by the breast cancer spheroids being 321 +/- 15 μm in diameter and the bladder cancer and colon cancer spheroids being the largest (514 +/- 17 μm and 628 +/- 17 μm in diameter, respectively). It has previously been shown that the size of the spheroids affects the sensitivity to electroporation with the smaller spheroids being most sensitive [14]. The fibroblast spheroids should therefore be more permeabilized and more affected than the cancer cell spheroids which are not the case (as described later and seen in Fig 1). Thus, in spite of the smaller size the fibroblast spheroids seem to be less sensitive to calcium electroporation than the cancer cell spheroids. It is also seen that the untreated normal cell spheroids decreased slightly in size over time indicating that these cells do not continue to grow over time as cancer cells. The breast cancer cell spheroids decreased in size over time likely because this is a metastasizing cell line, thus the cells easier detach from each other.

**Fig 2:**
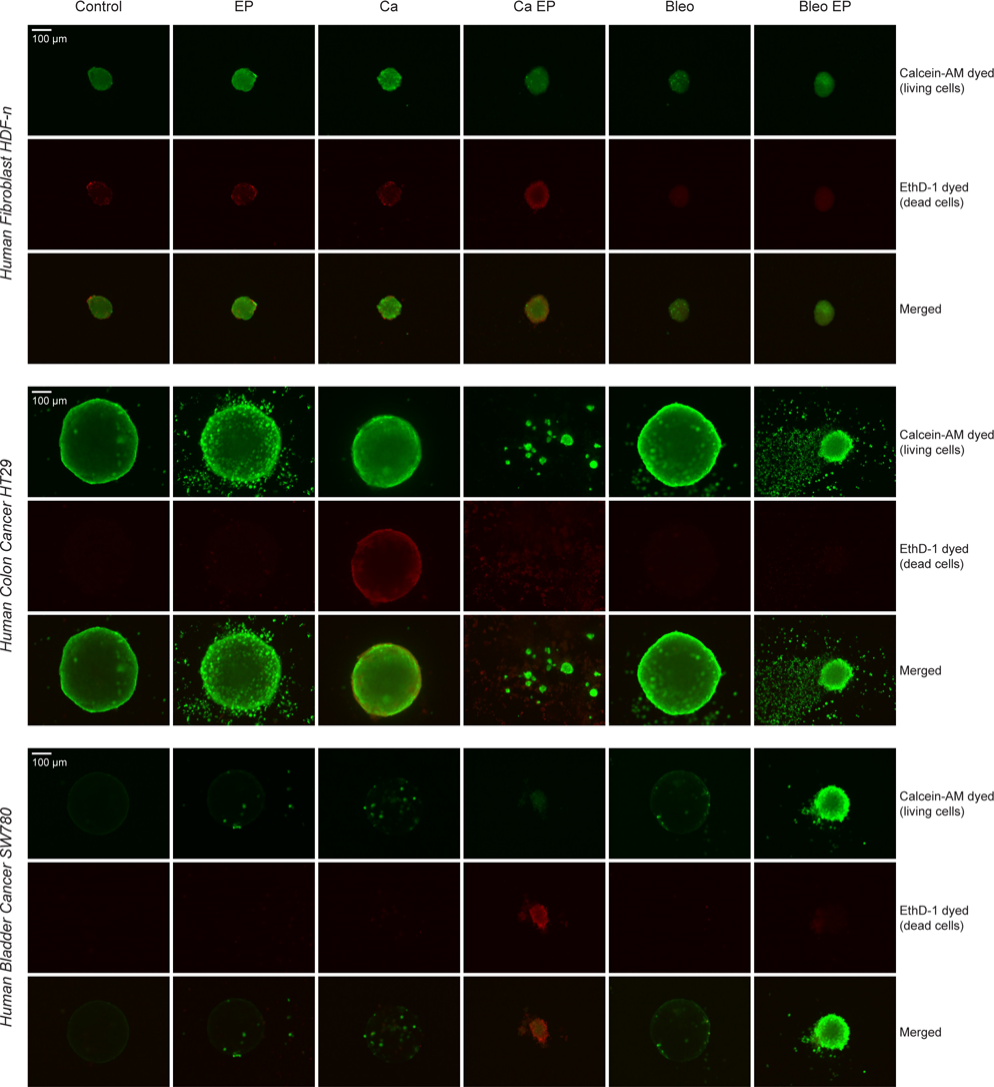
Live/dead staining. Live/dead staining with Calcein-AM and EthD-1 of human normal dermal fibroblast, colon cancer, and bladder cancer spheroids 4 days after treatment with 168 mM calcium (Ca), 1 mM bleomycin (Bleo), electroporation (EP; 8 pulses of 100 μs, 1000 V/cm, and 1 Hz), 168 mM calcium electroporation (Ca EP), or electrochemotherapy using 1 mM bleomycin (Bleo EP), and of untreated controls (Control). Upper panels are calcein-AM staining (living cells), middle panels are EthD-1 staining (dead cells), and lower panels are merged images of living (green) and dead (red) cells.

To investigate whether cells in the spheroids were dead or alive after treatment, the spheroids were examined using a viability/cytotoxicity assay where living cells were stained with calcein-AM and dead cells were stained with EthD-1 (Fig 2). The normal fibroblast spheroids stained with calcein irrespective of treatment, showing that many of the cells in these spheroids were still viable after the different treatments albeit with few cells staining EthD-1 indicating cell death. This could be due to the natural turn-over of these cells, and the amount of dead cells seems to be comparable in control as well as in treated spheroids. When looking at the colon cancer spheroids, it is clearly seen that there was a high death rate (low calcein staining and high EthD-1 staining) in the spheroids treated with calcium electroporation and electrochemotherapy using bleomycin. It is also seen that electroporation alone caused dissociation of some of the cells on the outer surface of the spheroid but did not kill the cells. Live/dead staining of the bladder cancer spheroids showed, similar to staining of that cell line with propidium iodide (Fig 1), that the dyes did not enter the spheroid. This could be due to tight junctions in the bladder cancer cell line.

Electrochemotherapy using bleomycin has a therapeutic window, where tumors are much more sensitive to electrochemotherapy than normal cells [17–19], thus the limited effect of electrochemotherapy using bleomycin on the normal fibroblast spheroids was expected. Interestingly, our present study could indicate that also treatment with calcium electroporation affects malignant and normal cells differentially.

**Fig 3:**
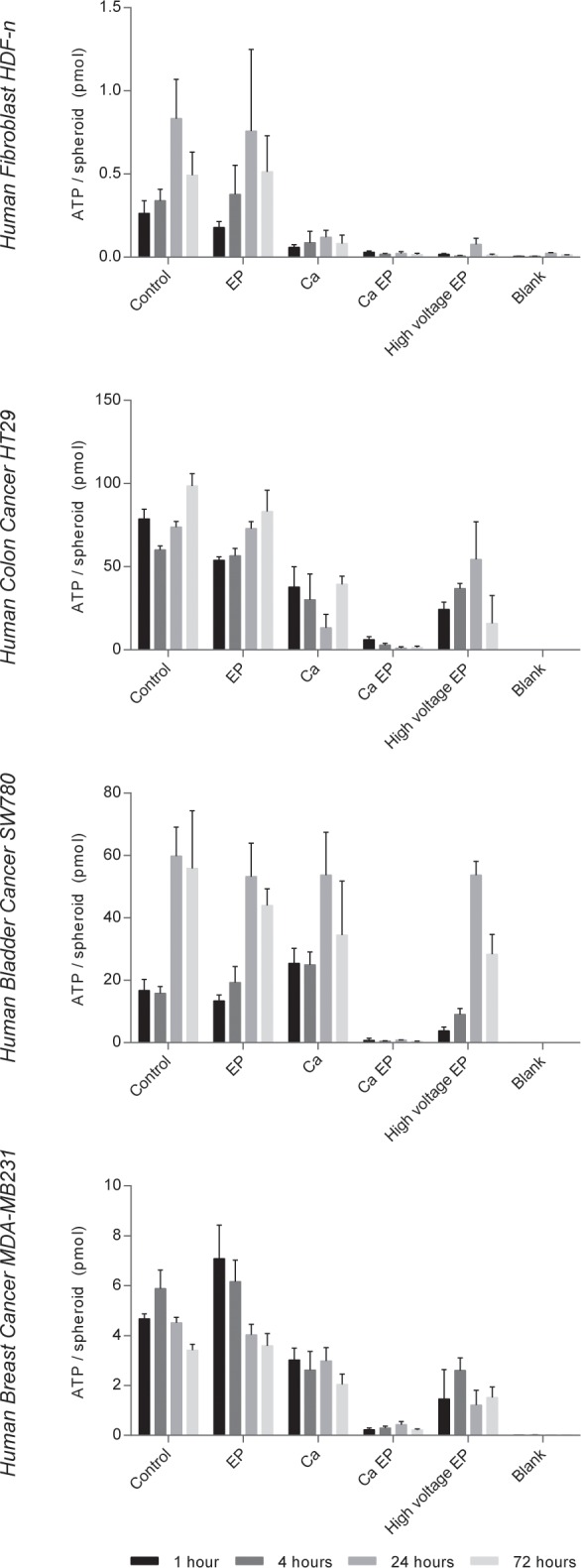
Intracellular ATP level. Intracellular ATP measurements of human normal dermal fibroblast, colon cancer, bladder cancer, and breast cancer spheroids 1, 4, 24, and 72 hours after treatment with 168 mM calcium (Ca), electroporation (EP; 8 pulses of 100 μs, 1000 V/cm, and 1 Hz), 168 mM calcium electroporation (Ca EP), high voltage EP (8 pulses of 100 μs, 5000 V/cm, and 1 Hz), and of untreated controls (Control). Means + SD, n = 3–5 (n = 2 for blank). Please note the difference in the y-axes.

ATP levels were previously shown to be acutely and severely depleted after electroporation alone as well as after calcium electroporation. The electroporated cells have re-established the ATP level 4 hours after treatment whereas in cells treated with calcium electroporation the ATP level stayed low for up to 8 hours after treatment [10]. The intracellular ATP level in the spheroids after calcium electroporation was measured (Fig 3) to investigate if the difference in sensitivity between cancer and normal cells could partly be explained by a difference in intracellular ATP level after treatment. Intriguingly, we found a dramatic decrease in ATP level both in cancer and normal cells after treatment with calcium electroporation (p<0.01). Thus, the effect of calcium electroporation on the intracellular ATP level cannot explain the difference in sensitivity; however the normal cells seem able to survive this decrease in ATP level whereas the highly metabolically active cancer cells do not. The amount of ATP per spheroid differs up to a 100 fold between the different cell spheroids likely due to the difference in size between the different spheroid types. In addition, we applied high voltage electroporation treatment, attempting to induce cell death. The intracellular ATP was actually not totally depleted in all these spheroids, indicating that all of the cells were not dead. Indeed, calcium electroporation significantly affected the intracellular ATP level more than treatment with high voltage pulses in the cancer cell spheroids (p<0.0001).

The electroporation parameters used in this study (8 pulses of 100 μs, 1000 V/cm (applied voltage to electrode distance ratio), and 1 Hz) is the same as the clinically used electroporation parameters. The linear array electrode is used in the clinic [20] and has 4 mm in distance between the electrodes exactly like the plate electrode used in this study. To test if the parameters caused permeabilization of the cells in the spheroid model, the spheroids were electroporated in the presence of propidium iodide (Fig 1). This showed that the cells were permeabilized using these parameters in accordance with previous work showing that all the cells within spheroids, even those in the center, were permeabilized after electric pulses application [15]. Cells in the center of the spheroids can be up to 19% smaller than the peripheral cells [21] and the different cell types differ in size, however cell size should not affect the sensitivity to treatment since all the cells in the spheroids were permeabilized (Fig 1). Interestingly, propidium iodide (668 Da) was not detected in all of the cells in the bladder cancer (SW780) spheroid. This could be explained by the presence of tight and adherent junctions in this epithelial bladder cell line impeding diffusion of propidium iodide into the center of the spheroid. Indeed, bladder epithelial cells are known to display tight junctions playing a critical role in the maintenance of a physiological, impermeable, urine-blood barrier [22]. This barrier prevents passage of ions and solutes between the urine and the blood. This could also explain the lack of live/dead staining in this spheroid type (Fig 2). However, the spheroids were clearly affected by calcium electroporation and electrochemotherapy using bleomycin indicating that these drugs, with electroporation, are able to enter the cells in the spheroid. The bladder cancer spheroids treated with calcium electroporation or electrochemotherapy using bleomycin showed more labeling after live/dead staining than spheroids treated with the other conditions. It could be that electroporation with these drugs loosens or alters tight junctions, however more research is needed. Indeed, bladder cancer is a likely novel target for electrochemotherapy or calcium electroporation [23,24].

Electrochemotherapy using bleomycin is used in the clinic for treatment of cutaneous metastasis of all histologies [5,13,18,25]. Calcium electroporation is currently being investigated in a double-blinded randomized clinical trial, where intratumoral injection of either bleomycin or calcium followed by electroporation is evaluated for response. It has previously been shown that when treating cutaneous metastasis with electrochemotherapy the surrounding normal tissue is much less affected [18]. However, the effect of calcium electroporation on normal cells has not been investigated. Here, we have shown that both calcium electroporation and electrochemotherapy using bleomycin effectively induce cell death in the tested cancer cell spheroids but the treatments do not seem to affect the normal fibroblast spheroids to the same degree even though a similar loss of intracellular ATP level was observed in all cell lines. Reasons for this apparent discrepancy in sensitivity to calcium electroporation between malignant and normal cells could be differences in calcium signaling pathways [26], calcium channel expression [27], cell death pathways [28], and other phenomena e.g. effects on the microtubule system [29] or membrane repair system [30]. The discrepancy could also be due to difference in the active calcium transport [31,32] such as the expression of the plasma membrane calcium ATPases. Further investigations are needed to understand the cell death pathway of calcium electroporation and the difference in sensitivity.

The perspectives for calcium electroporation are many since electroporation based treatments are increasingly used to treat cutaneous metastases [5,13], in guidelines for treatment of cutaneous tumors [33], and also increasingly used for tumors in internal organs e.g. for liver, brain, and bone metastases [2,4,34]. Several clinical trials are ongoing for irreversible electroporation in treatment of tumors in internal organs [35–38] and the treatment field in irreversible electroporation may be enlarged using calcium as an adjuvant. Furthermore, calcium electroporation may be of particular interest since it can be used directly by interventional radiologists and surgeons since oncologists are not required for application of this novel treatment.

In conclusion, calcium electroporation is a novel, efficient, simple, and inexpensive procedure, showing very promising results in preclinical studies, and is currently in clinical trial. The finding of a differential effect to calcium electroporation in malignant and normal cell spheroids is promising and further research is needed to verify this on other normal cell types as well as *in vivo* and in patients to validate the findings and to understand the mechanism behind this difference.
